# Cardiorenal Fat: A Cardiovascular Risk Factor With Implications in Chronic Kidney Disease

**DOI:** 10.3389/fmed.2021.640814

**Published:** 2021-05-25

**Authors:** Luis D'Marco, María Jesús Puchades, Nayara Panizo, María Romero-Parra, Lorena Gandía, Elena Giménez-Civera, Elisa Pérez-Bernat, Miguel Gonzalez-Rico, José Luis Gorriz

**Affiliations:** ^1^Nephrology Department, Hospital Clínico Universitario, Institute of Health Research (INCLIVA), Valencia, Spain; ^2^Universidad de Valencia, Medicine School, Valencia, Spain

**Keywords:** chronic kidney disease, adipose tissue, cardiovascular disease, cardiorenal disease, renal failure

## Abstract

There is a growing interest in the potential role of adipose tissues in cardiac and renal pathophysiology, and determining the mechanisms by which fat compartments around the heart and kidneys influence cardiovascular disease is of clinical importance in both general and high-risk populations. Epicardial fat and perirenal fat have been associated with adverse outcomes in chronic kidney disease (CKD) patients. Epicardial fat is a rich source of free fatty acids and is capable of secreting inflammatory and pro-atherogenic cytokines that promote atherosclerosis through a local paracrine effect. Recent evidence has demonstrated that perirenal fat has a closer correlation with kidney diseases than other visceral fat deposits in obesity or metabolic disturbances. Moreover, perirenal fat has been reported as an independent risk factor for CKD progression and even associated with cardiorenal dysfunction. Accordingly, these forms of organ-specific fat deposits may act as a connecter between vascular and cardiorenal disease. This review explores the possible links between epicardial and perirenal fat and its significant role as a modulator of cardiorenal dysfunction in CKD patients.

## Introduction

Interest in the potential impact of organ-specific adipose tissue on cardiac and renal pathophysiology is growing, and it is clinically important to determine the mechanisms by which fat compartments around the heart and kidneys influence cardiovascular disease (CVD) in both general and high-risk populations (1, 2). In these sense, epicardial fat (EF) and perirenal fat (PF) have been associated with adverse outcomes in chronic kidney disease (CKD) patients (3–5). EF and PF tissues accumulate with overweight and obesity (6), which are associated with microalbuminuria (7, 8), and thus with worse prognosis in CVD patients (9).

The close anatomical proximity and intercommunication between EF, coronary arteries and myocardium has been reported as a key factor in atherosclerotic disease development and progression (10). As a rich source of free fatty acids capable of secreting inflammatory and pro-atherogenic cytokines, EF is believed to promote atherosclerosis of the intra-epicardial branches of coronary arteries through a local paracrine effect. Interestingly, local secretion of certain adipocytokines in inflamed peri-coronary adipose tissue may have adverse consequences on myocardial contractility and vascular calcification (11). PF, the adipose tissue surrounding the kidneys, was originally thought to provide only mechanical kidney support, yet studies have shown have a closer association of PF with kidney diseases than other visceral fat deposits in obesity or metabolic disturbances (12). PF has recently been identified as an independent risk factor for CKD progression and is even associated with cardiorenal dysfunction (13). Finally, these forms of organ-specific fat deposit may act as a connector between vascular and cardiorenal disease. This review outlines the evidence of possible links between epicardial and PF and its significant role as a modulator of cardiorenal dysfunction under pathologic conditions in patients affected by renal disease.

## Epicardial FAT

EF represents adipose tissue located below the visceral pericardium in direct contact with the epicardial coronary arteries (14). EF originates in the splanchnopleuric mesoderm, without fascia separating it from myocardium and arteries, which allows a direct flow of fatty substances to the coronary arteries and myocardium (15). Moreover, EF can also be found amid myocardial fibers, generally next to the intra-myocardial branches of the coronary arteries (16, 17).

Although the functional role of EF is complex and currently remains undefined, this tissue participates in multiple areas, such as mechanical, metabolic, thermogenic and endocrine/paracrine/autocrine functions (18). EF accompanies the principal branches of the coronary arteries along their atrioventricular course to provide mechanical protection against excessive deformation during the cardiac cycle (19). Moreover, EF is actively involved in energy production and lipid homeostasis, and has a higher rate of free fatty acid (FFA) release and absorption than other fat deposits (18). Since myocardial metabolism is dependent on FFA oxidation, EF supports myocardial energy needs, especially during periods of high demand (19). Like PF, brown adipose tissue is rich in mitochondria with significant production of the uncoupling protein-1 (UCP1) needed to produce heat in response to cold exposure. Evidence has shown that expression of UCP1 and related genes is higher in EF and other fat deposits such as abdominal or subcutaneous adipose tissue (20). These findings indicate a role for EF in heat production to protect the heart from hypothermia.

Studies in animals and humans have shown that EF secrete an abundance of cytokines (Table 1), which are implicated in control of endothelial function, coagulation, and inflammation (11, 16). These numerous bioactive molecules produced by EF can protect or negatively affect the myocardium and coronary arteries (21). Under physiological conditions, EF secretes cytokines with anti-inflammatory and anti-atherosclerotic properties, such as adiponectin and leptin (22). Secreted by adipocytes, adiponectin has antioxidant, anti-inflammatory and anti-atherogenic actions (23): it stimulates the oxidation of fatty acids *via* a protein kinase pathway to reduce lipid storage in the myocardium, and also inhibits production of inflammatory mediators to maintain an anti-inflammatory environment in the cardiovascular system. Conversely, under pathological conditions adiponectin production by EF drops, while production and secretion of pro-inflammatory or pro-atherogenic cytokines (such as leptin, vinfatin, or apelin) are increased (11, 21). Studies have linked leptin with pro-atherogenic effects, hypertension, endothelial dysfunction, inflammation, oxidative stress, and proliferation of vascular smooth muscle cells (24, 25), yet surprisingly, adipocyte-derived factors such as adiponectin and leptin remain controversial in the setting of renal dysfunction.

**Table 1:** Pathophysiological effects of adipocytokines produced in organ-specific adipose tissue.

**Adipokine**	**Metabolism in CKD**		**Cardiorenal effects**					
	**Accumulation**	**Outcome**	**Oxidative stress**	**Ischemia/reperfusion**	**LV hypertrophy**	**Remodeling**	**Inflammation**	**Proteinuria**
Adiponectin	Yes	Inflammation/CVD	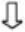	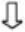	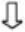	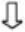	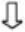	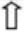
Leptin	Yes	Inflammation/CVD	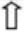	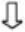	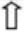	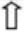	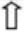	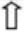
Visfatin	Yes	Endothelial damage/lipid dysregulation/CVD	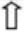	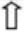	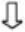	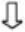	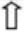	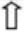
Apelin	Yes	Inflammation/CVD	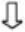	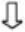	U	U	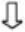	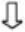
Resistin	Yes	Endothelial damage/inflammation/CVD	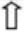	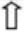	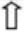	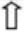	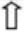	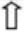
Omentin	Yes	Endothelial damage/inflammation/CVD	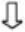	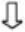	U	U	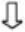	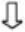

*CKD, chronic kidney disease; CVD, cardiovascular disease; LV, left ventricle; U, unknown. Adapted from D'Marco et al. (11)*.

## Perirenal FAT

PF represents fat located in the retroperitoneal space surrounding the kidneys. Renal sinus fat is also a component of PF and this adipose tissue is surrounded by fascia tissue. Regardless of its pre-adipocyte origin, PF undergoes an unusual progressive transition from brown to white adipose tissue after birth (26). In adults, PF is composed of a combination of white and brown adipose tissue (27). Anatomically, this fat is extensively vascularized by abdominal aorta branches including the inferior adrenal, dorsal, and gonadal arteries (28).

Research has focused on the connections between the kidneys and PF, as well as between this tissue and bodily function. As observed in EF, PF produces and secretes adipokines and pro-inflammatory cytokines, including adiponectin, leptin, visfatin, resistin, tumor necrosis factor-α and interleukin-6 (IL-6), among others (13, 29) (Table 1). Moreover, these substances pass near to the kidneys and assist in regulating renal function through endocrine and paracrine pathways (30). PF has therefore been linked to direct lipotoxic effects such as increasing hydrostatic pressure in the glomerulus, activating the renin-angiotensin-aldosterone system, and accelerating CKD progression (31, 32). In this regard, an association between PF and abdominal obesity and albuminuria has been detected in obese patients (33, 34). Although studies have reported an association of PF with CKD progression, new evidence on the presence and accumulation of PF is warranted in this risk group (35, 36).

## Organ-Specific FAT Measurement Techniques

Several non-invasive techniques have been used to measure body fat distribution, especially *via* computed tomography (CT) and magnetic resonance imaging (MRI) (37, 38), although their use is hindered by the high cost and exposure to radiation. Studies have demonstrated that ultrasound is a practical and reasonable alternative to CT or MRI, given that this imaging technique involves no radiation exposure and is cheap and reproducible (39). Other studies have explored the use of abdominal ultrasound to determine PF burden (40). EF has also been measured with echocardiography techniques; however, these imaging methods offer only a partial approach to organ-specific adipose tissue, since measurement is limited to tissue (41). In contrast, CT and MRI techniques allow more spatial resolution with extended measures such as the total volume of fat surrounding these organs. Finally, although DEXA and Bioimpedance techniques offer interesting quantification of total body fat, as yet no specific organ compartments can be measured by these devices.

## Organ-Specific FAT as Causative Factor of Cardiorenal Disease

**Figure 1:**
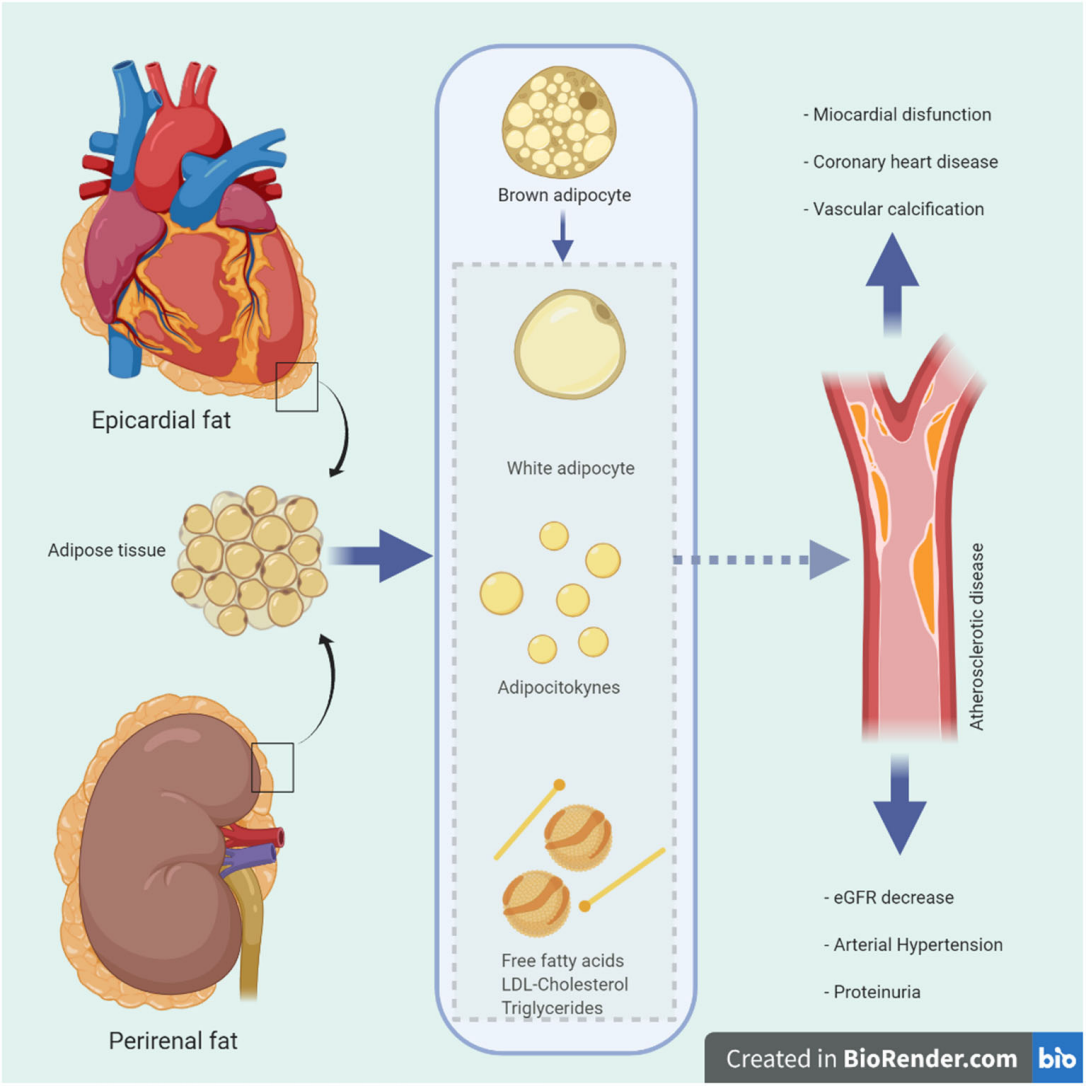
Possible physiological and pathological mechanisms of epicardial and renal fat in vascular beds. Schematic diagram illustrating the anatomical location of epicardial and perirenal fat around the heart and kidneys, its various pathophysiological functions and proposed pathways of damage with adverse clinical consequences. eGFR, estimated glomerular filtration rate; LDL, low density lipoprotein.

Recent evidence has shown that region-specific adipose tissue is an important cardiovascular risk marker in the general and high-risk population. For example, there appears to be a connection between EF and CVD in conditions as common as high blood pressure, metabolic syndrome, diabetes, and cardiorenal disease (31, 32). Recognition of the adipocyte as a cell type with complex functioning including endocrine, paracrine and autocrine capacity has aroused research interest in disciplines such as nephrology, cardiology, and endocrinology, and it has become a fertile ground for scientific debate. Its wide heterogeneity of location, functions, proteomics, and metabolomics (1) are particularly important in adiposity, where changes in function and volume are associated with increased cardiorenal and metabolic risk (Figure 1).

Although studies evaluating EF in CKD are still ongoing, some research has reported a relationship between EF and vascular disease in patients with renal involvement (42, 43) (Table 2). In a study carried out in a cohort of more than 400 patients with CKD stage 4-5 and 5 on dialysis EF emerged as a risk factor for alterations in myocardial perfusion as well as appearance of vascular calcification (3), and was also independently associated with vascular calcification in predicting myocardial perfusion defects in this study. Karatas et al. recently demonstrated that EAT thickness measured by echocardiography was significantly greater in hemodialysis patients than in those in pre-dialysis stages or in healthy subjects (47). Similarly, in a sub analysis of the RIND study (Renagel in a new dialysis trial), which initially evaluated the presence of coronary artery calcifications (CAC) and alterations in calcium and phosphorus in patients starting dialysis treatment, it was observed that each 10 cc increase in EF volume raised mortality by 6% (4). Other factors such as CAC and age were also associated with increased mortality. Further publications have reported that patients with CKD have higher EF thickness and volume compared to control subjects (50). Conversely, in other research EF was not confirmed as an isolated risk predictor but rather as a cofactor associated with body mass index (BMI) and other obesity indices.

**Table 2:** Different studies showing epicardial and perirenal fat with different outcomes in CKD populations.

**References**	**Technique**	**Study population**	***N***	**Outcomes**
**Perirenal Fat**				
Shen et al. (34)	Ultrasound	T2DM patients with Albuminuria	89	Increased PF was associated with albuminuria in patients with T2DM
D'Marco et al. (5)	Ultrasound	CKD patients in all stages (non-dialysis)	103	PF thickness was associated with metabolic risk factors in CKD patients
Fang et al. (35)	Ultrasound	Patients with T2DM and reduced eGFR	171	This study confirmed a negative independent relationship between PF and eGFR in patients with T2DM
Geraci et al. (36)	Ultrasound	Hypertensive patients	216	PF significantly correlated with eGFR with no differences in groups divided by sex, diabetes, or BMI.
Lamacchia et al. (44)	Ultrasound	T2DM subjects	151	PF was an independent predictor of eGFR decrease and altered renal resistance index and hyperuricemia
Koo et al. (45)	CT	Asymptomatic subjects, hypertension and dyslipidemia.	3.919	PF was independently associated with vascular calcifications.
**Epicardial fat**				
Turkmen et al. (43)	CT	ESKD patients on dialysis + 27 healthy subjects.	80	EF significantly correlated with MIA syndrome in ESKD patients
Karohl et al. (3)	CT	CKD stages 4-5 patients awaiting kidney transplantation	411	EF was the best predictor of abnormal myocardial perfusion in CKD patients.
D'Marco et al. (4)	CT	HD patients	95	Each 10 cc increase in EF volume was associated with a significant 6% increase in risk of death during follow-up
Erdur et al. (42)	CT	ESRD patients on dialysis + 42 healthy subjects	76	Age and BMI are independent predictors of EF.
Ko et al. (46)	CT	HD patients	109	EF progression slows significantly with non-calcium-based resins (Sevelamer)
Karatas et al. (47)	Echocardiography	CKD and HD patients + healthy subjects	111	EF is significantly high in HD patients compared to pre-HD and healthy subjects.
Ayan et al. (48)	Echocardiography	CKD all stages, dialysis, and healthy subjects	97	No correlations between EF thickness and inflammatory markers were found.
Cano Megías et al. (49)	CT	CKD and HD patients	104	A higher EF was associated with increased mortality and lower free survival time to fatal and non-fatal cardiovascular events

*T2DM, type 2 diabetes mellitus; PF, perirenal fat; CKD, chronic kidney disease; eGFR, estimated glomerular filtration rate; BMI, body mass index; CT, computer tomography; ESKD, end stage kidney disease; EF, epicardial fat; MIA, malnutrition inflammation atherosclerosis; HD, hemodialysis*.

Recent studies have identified excess PF as an emerging risk marker of CVD, independently of common metabolic risk factors (13, 44). Our previous analysis showed that PF thickness was associated with metabolic risk factors in non-dialysis-dependent patients (5). Other investigations found PF thickness to be positively correlated with high blood pressure in overweight patients and hypertension (6, 51). Moreover, higher levels of PF have been linked with carotid intima-media thickness, indicating that excess PF is associated with atherosclerosis and CVD (52).

As a CVD-related risk factor, diabetes or dyslipidemia-associated PF may also indirectly contribute to CVD. A recent study showed that excess PF was independently associated with hyperinsulinemia and insulin resistance in obese patients, irrespective of other anthropometric or metabolic parameters, suggesting PF as a strong marker of insulin resistance (6). Moreover, PF development and remodeling have also been associated with other metabolic parameters in patients with CKD. Hence, PF thickness was significantly correlated with abnormal triglycerides and uric acid levels, in which patients with stage 4 and 5 CKD had the greatest PF thickness (5). Of interest, excess PF is associated with a reduced glomerular filtration rate, regardless of other indices of adiposity, in patients with hypertension and or diabetes (36). A more recent study reported a positive correlation between PF and albuminuria among type 2 diabetes patients (35).

The physio-pathological processes and association of these specific cardiac and renal adipose tissues allow the hypothesis that an excess of epicardial and PF contributes to CVD. PF is potentially related to EF as both exhibit mesothelial layers like visceral organs enriched in white adipose tissue progenitors which produce adipocytes (12, 53). This finding bolsters the hypothesis of PF as a risk predictor for CVD in the renal-affected population, and likewise, EF has been considered a CVD risk factor that predicts cardiac dysfunction in CKD patients (48, 49). Regarding this latter proposal, these adipose tissues are linked to proven cardiovascular indicators such as carotid intima-media thickness and vascular calcifications indexes (4, 45, 52), and they also express browning fat markers with interactions between both tissues through complex endocrine pathways with differential behaviors to those observed in other adipose tissues (54–56).

## Adipose Tissue and Bone Mineral Axis with Cardiorenal Implications

Elevated serum levels of fibroblast grow factor 23 (FGF23) are strongly associated with increased CVD in CKD patients. Hence, FGF23 levels rise as kidney function declines, with the result that in patients with end state kidney disease (ESKD), FGF23 contributes to hypertension, vascular calcification, and left ventricular hypertrophy by distinct mechanisms (57). It is currently accepted that adipocytes are capable of storing vitamin D, and beyond this activity new evidence has revealed crosstalk between energy storage organs such as adipose tissues and bone. Recent reports show that adiponectin and leptin directly stimulate FGF23 expression in the skeleton (58), thus suggesting a link between these adipokines and FGF23. Moreover, a recent investigation found adiponectin to be a strong modulator of FGF23 response to vitamin D receptor (VDR) activation in CKD patients (59). Of note, mice overexpressing the most abundant adipose tissue protein, adiponectin, exhibit a substantially enhanced FGF23 response to phosphate load compared with wild-type and adiponectin knock-out mice. Additionally, VDR activation markedly upregulates FGF23 gene expression and substantially increases circulating levels of FGF23 in healthy subjects with vitamin D deficiency and CKD patients. Accordingly, the association between FGF23 and fat mass was correlated with higher serum leptin levels in animal models, which adds support for leptin regulation of FGF23, and thus FGF23 was also associated with cardiac and endothelial damage (60). Beyond the correlation between EF and coronary calcification, insulin resistance and inflammation (IL-6), FGF23 has showed an association with EF in stage 3-5 CKD patients (61).

We postulated that local peri-vascular expression of adiponectin and leptin may dysregulate local FGF-23 production in calcified endothelial cells, suggesting the possibility of an undercover regulatory pathway in a uremic environment. This mechanism may explain why EF has been reported as an independent risk factor for CAC and myocardial dysfunction in CKD patients (3).

## Therapeutic Approaches

**Figure 2:**
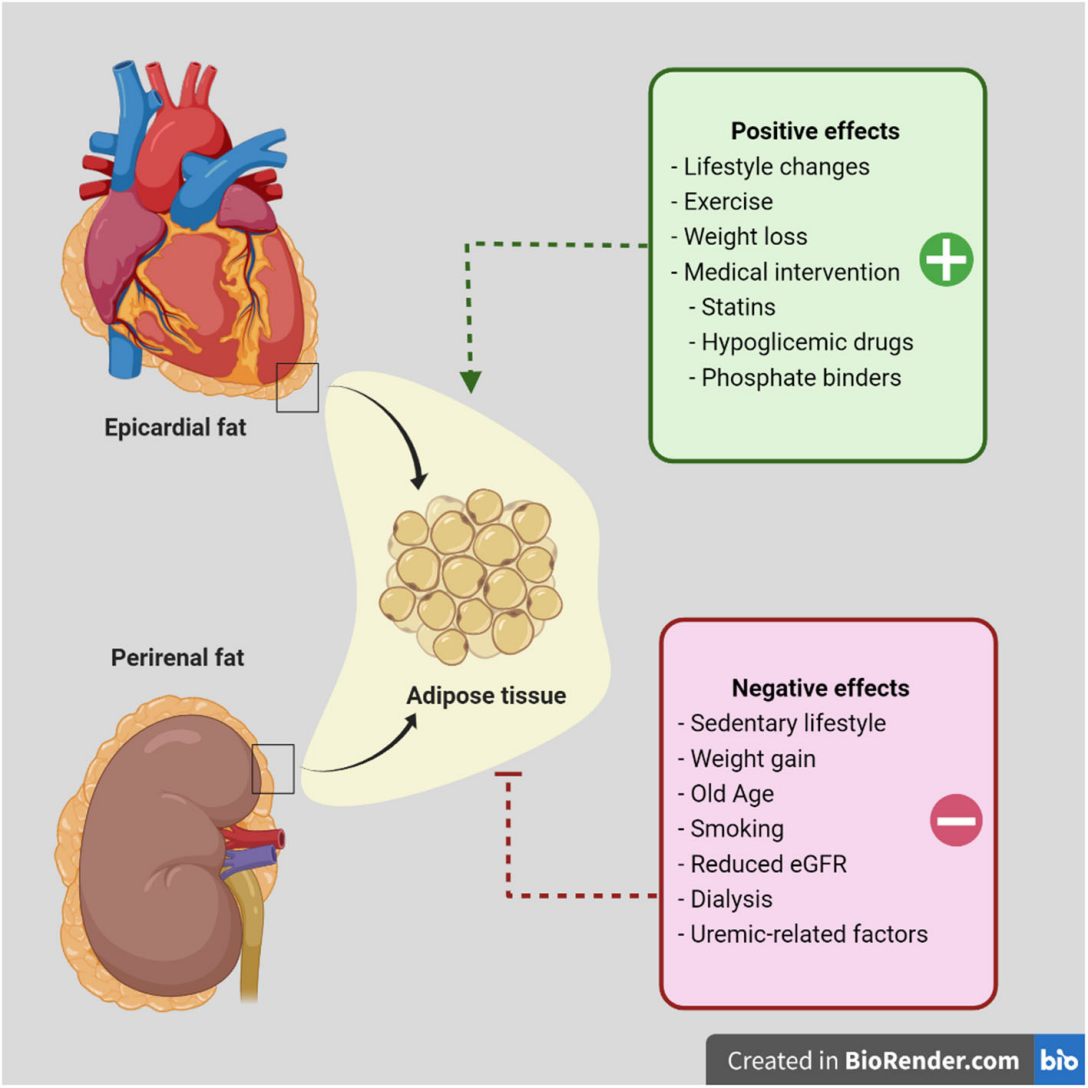
Proposed factors that improve or worsen epicardial or perirenal fat tissue accumulation in patients with chronic kidney disease. Diagram showing adipose tissue around the heart and kidney. In a pathological environment, this adipose tissue becomes a cardiovascular risk factor in susceptible populations. Current evidence has been published regarding the effect of lifestyle changes and weight loss or exercise. Moreover, medical intervention has supported the use of hypoglycemic agents and other drugs to reduce adipose tissue accumulation and/or dysfunction. Although further evidence regarding perirenal fat intervention is warranted in future studies, controlling metabolic abnormalities such as hyperglycemia, dyslipidemia or hyperuricemia may improve clinical outcomes.

Organ-specific adipose tissue reduction has been achieved with lifestyle changes and pharmacological interventions (Figure 2). Intriguingly, a study showed a 2% regression in EF volume in patients who lost 5% of their body weight, compared to a 23% increase in those who gained weight (62). Moreover, other reports describe decreased EF volume after intensive lipid-lowering therapy with statins (63, 64). This effect may be related to the known anti-inflammatory activity and inhibition of vasa-vasorum proliferation shown by statins (65).

In a recent study, Couselo-Seijas et al. showed that treatment with sodium-glucose co-transporter 2 inhibitor (SGLT2i) reduced EF (66), and use of an SGLT2i (Dapagliflozin) thus improved glucose metabolism with no lipogenesis-involved gene regulation or lactate production, mainly in patients with CAC. Interesting findings from another study provide insights into the distinctive role of the PPARα/adiponectin/SGLT2 pathways in regulating sodium and glucose homeostasis in PF (67). This work showed that excessive sodium intake also increased plasma level of adiponectin, as well as its expression in both PF and renal cortex. Other research indicated that glucagon-like peptide-1 (GLP-1) analogs Exenatide and Liraglutide are effective in reducing EF in obese patients with diabetes mellitus, effects that are mostly weight-loss dependent (68). In diabetic patients, adding a dipeptidyl peptidase-4 (DPP-4) inhibitor to standard care therapies produced a significant decrease in EF volume over 2-year follow-up (41). Similar benefits have been observed with pioglitazone in patients with metabolic syndrome (69).

In patients with ESKD, use of non-calcium-based phosphate binding agent sevelamer reduced serum cholesterol and markers of inflammation (70). Additionally, sevelamer has shown to be effective in slowing down CAC progression and EF in dialysis patients (46). The effect of weight loss and regular exercise on accumulation of visceral adipose tissue in CKD patients remains to be elucidated.

## Conclusions

Renal-affected patients express extremely high incidence of CVD usually attributed to classical and uremic-related conditions. This suggests that specific adipose tissues may represent an additional cardiovascular risk factor in CKD patients. Finally, organ-specific adipose tissue, specifically EF and PF, represents an interesting and underexplored field to consider in this population.

## Author Contributions

All authors listed have made a substantial, direct and intellectual contribution to the work, and approved it for publication.

## Conflict of Interest

The authors declare that the research was conducted in the absence of any commercial or financial relationships that could be construed as a potential conflict of interest.
